# An updated immunosenescence exploration in healthy Chinese donors: circular elevated PD-1 on T cell and increased Ki67 on CD8+ T cell towards aging

**DOI:** 10.18632/aging.205985

**Published:** 2024-06-29

**Authors:** Yue Chang, Wei Cao, Lianfeng Lu, Yang Han, Lin Qin, Baotong Zhou, Taisheng Li

**Affiliations:** 1 Department of Infectious Diseases, Peking Union Medical College Hospital, Chinese Academy of Medical Science and Peking Union Medical College, Beijing, People’s Republic of China; 2 School of Clinical Medicine, Chinese Academy of Medical Science and Peking Union Medical College, Beijing, People’s Republic of China; 3 State Key Laboratory of Complex Severe and Rare Diseases, Peking Union Medical College Hospital, Chinese Academy of Medical Science and Peking Union Medical College, Beijing, People’s Republic of China; 4 Tsinghua University Medical College, Beijing, People’s Republic of China

**Keywords:** immunosenescence, immunophenotyping, lymphocyte subsets, flow cytometry, aging

## Abstract

Immunosenescence is a process of immune dysfunction that occurs along with aging. Many studies have focused on the changes of different lymphocyte subsets in diseases and immune aging. However, the fluctuation in the number and phenotype of lymphocyte subset caused by aging have not been comprehensively analyzed, especially the effects of new indicators such as PD-1 and Ki67 in peripheral blood have been rarely reported. We further investigated the humoral and cellular immune parameters of 150 healthy donors over 18 years old. Age was associated with decreased CD4+CD45RA+CD62L+ T cells, decreased CD4+CD45RA+CD31+ T cells, and increased memory CD4+ or CD8+ T cells, dominated by male CD8+ T cells. The loss of CD28 expression on T cells and the transverse trend of activated CD38 and HLA-DR were also related to the increased age. In addition, CD8+ T cells in men were more prominent in activation indicators, and the difference between the old and young groups was obvious. CD4+CD25+CD127- T cells percentage tended to decrease with age and did not differ significantly between gender. Interestingly, we found that age was positively associated with PD-1+ T cells and showed significant age-related variability in men. Similarly, the percentage of CD8+ki-67+ also showed an increasing trend, with significant differences between the young group and other elderly groups in males. Our findings can provide immunological clues for future aging research, offering new insights for clinical monitoring and prevention of certain diseases.

## INTRODUCTION

Immunosenescence is a ubiquitous remodeling of immune functions which involves both adaptive and innate immunity. It is a well-known risk factor for cancer development, with incidence increasing disproportionately with age. [1, 2]. The evolution of cellular immune parameters during the lifetime in healthy population has been intriguing researchers worldwide recently, but the published conclusions vary tremendously [3]. The robust trends of peripheral lymphocyte phenotype coming along with aging are still pending. Our understanding of the mechanisms driving age-related immune system decline, immune dysfunction during the aging process, and the impact of the elderly immune system on age-related diseases remains incomplete.

Research in aging model systems has shown significant changes in the function and phenotype of T cells with increasing age [4]. To fulfill the health needs of the growing elderly population, research on age-related immune senescence needs to progress rapidly. Adaptive immunity of T cells has always been a primary focus of research on immunosenescence [1, 2, 4, 5]. Mittelbrunn and Kroemer recently proposed 10 hallmarks of T cell aging [6], including phenotypic changes such as naïve-memory T cell imbalance, reduced T cell receptor (TCR) pool, T cell senescence and lack of effect plasticity. Several researchers have investigated the changes in NK cells during the aging process [6–8]. However, the controversial results may attribute to study population and control groups selected in these studies [9, 10]. This process is also associated with a decrease in initial B cell production and an increase in the pool of low-clone B cells, resulting in altered reactivity to novel antigens. The characteristics of immunosenescence include a decline in cell-mediated immune function and humoral immune responses [3, 11].

As early as 2016 [9], our team conducted an observational study of immune parameters among 1,068 healthy adults, which demonstrated elementary clues for exploring immunosenescence, involving development, activation, and differentiation. In the state of chronic infections, cancer, and other diseases, the immune system undergoes aging due to prolonged exposure to external/viral antigen stimulation, accompanied by cellular exhaustion or abnormal proliferation, resulting in the accumulation of dysfunctional, terminally differentiated cells [12, 13]. However, the age trend of some emerging lymphocyte subsets such as PD-1 and Ki67 remains unclear, which have played an indispensable role in assessing immune status and clinical diagnosis. Especially in the elderly population, changes in the phenotype and activity of their immune cells lead to impaired immune function, affecting their health status. In this study, we aim to depict age-related fluctuations in peripheral blood lymphocyte subpopulations, providing reference for research on immune aging and various diseases.

Given previous research, we further evaluated the gender differences in immune aging and the associated phenotypic changes in lymphocyte subsets, including markers PD-1 and Ki67 reflecting peripheral blood exhaustion and proliferation function. It provides a rich perspective on immune aging in the elderly, offering a systematic reference for immune status analysis and clinical diagnosis and treatment.

## MATERIALS AND METHODS

### Subjects

A cross-sectional study was conducted between June and September 2022 among 150 healthy adults, including 90 males and 60 females, aged over 18 years. Subjects testing positive to HIV, systemic infection, connective tissue disease, abnormal tumor marker or cancer were excluded. According to the defined criteria from the SENIEUR protocol guideline [14], subjects were classified as older (≥65 years), middle-aged (45–64 years), and younger (18–44 years). All subjects received informed consent, and the ethics committee of Peking Union Medical College Hospital approved the study (Ethics number: I-23PJ463).

### Lymphocyte count and phenotyping analysis

**Figure 1:**
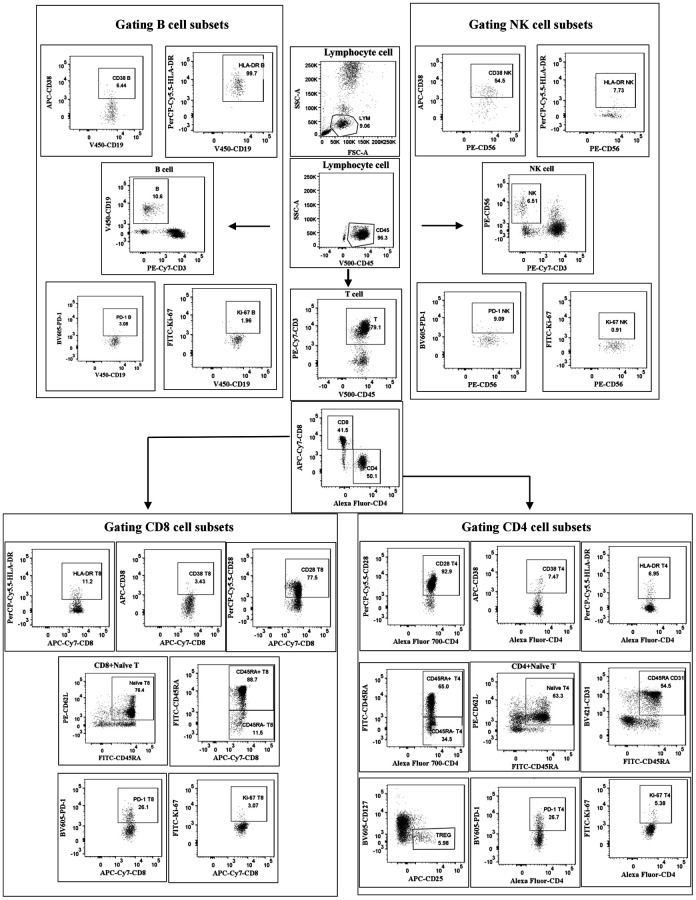
**Gating strategy.** Cells were first gated for lymphocytes (SSC-A vs. CD45), then CD3+ T cells, CD4+ T cells, CD8+ T cells, CD56+ NK cells and CD19+ B cells were positively determined. Next, the subtle subsets underlying each type of cell were further identified and differentiated.

Fresh EDTA-anticoagulated whole blood was collected and treated by flow cytometry within 6 h. Eighteen color flow cytometry (LSRFortessa and trade; BD Biosciences, La Jolla, CA, USA) analyzed peripheral blood lymphocyte subsets. Isolated PBMC from whole blood was incubated and tested with a panel of monoclonal antibodies against: anti-CD56-PE/anti-PerCP-cy5.5-HLA-DR/anti-CD38-APC/anti-CD3-PE-cy7/anti-CD8-APC-cy7/anti-CD4-AF700/anti-CD19-V450/anti-CD45-V500C/anti-PD-1 BV605/anti-CD45RA-FITC/anti-CD62L-PE/anti-CD28-PerCP-cy5.5/anti-CD25-APC/anti-CD127-V450/anti-CD31-BV421. Antibodies for this study were purchased from BD Pharmingen (San Diego, CA, USA). The samples were analyzed using FACSDiva software (Becton Dickens, Franklin Lakes, NJ, USA). The gating strategy for lymphocyte subsets is shown in Figure 1. Cell counts for lymphocyte subsets were calculated using a two-platform approach, where white blood cell counts and lymphocyte differentials were obtained from routine blood tests of the same specimen.

### Statistical analysis

All data parameters were subject to normality testing using the Shapiro–Wilk normality test. The continuous variables were described as mean or median and compared by Kruskal–Wallis Rank Sum Test or Mann–Whitney *U*-test when data did not conform to a normal distribution. Probability value was obtained from two-sided tests and *P* < 0.05 were considered significant. Statistical analysis was performed with SPSS software (SPSS^®^ for Windows™ version 21.0, SPSS Inc, Chicago, IL, USA) and GraphPad Prism software (GraphPad Software^®^ for Windows™ version 9.0, Boston, MA, USA).

## RESULTS

### The characteristics of study population

A total of 150 healthy Chinese adults were recruited in this study, including 90 men (60.0%) and 60 women (40.0%) with age spanning from 19 to 83. In this study, the 95% confidence interval recommended by the International Committee for Clinical Laboratory Standardization was used to determine the detailed distributions of lymphocyte subsets by the combination of normal distribution method and percentile method, as shown in Table 1.

**Table 1:** Peripheral blood lymphocyte subset reference values.

**Parameters**		**Mean**	**Median**	**Std. deviation**	**Range**	**Min–Max**
Lymphocyte	(%)	26.2	29.7	5.4	20.6–35.8	20.6–52.7
	(cells/μl)	1936	1901	466	1551–2321	950–2947
CD19+ B	(%)	9.96	10.00	3.8	7.0–12.0	2.2–22.7
	(cells/μl)	190	186	80	131–247	18–434
CD16+CD56+ NK	(%)	16.6	15.0	6.8	12.0–21.2	2.1–37.0
	(cells/μl)	324	301	162	205–403	36–789
CD3+ T	(%)	69.1	70.8	8.5	62.3–75.5	44.9–86.5
	(cells/μl)	1338	1292	362	1091–1553	509–2260
CD3+CD4+/CD3+	(%)	52.5	51.9	10.5	44.3–60.1	27.7–77.5
	(cells/μl)	702	644	250	525–842	233–1506
CD3+CD8+/CD8+	(%)	37.9	37.5	9.6	32.3–45.0	16.0–65.3
	(cells/μl)	506	485	187	358–609	95–1013
CD4+/CD8+	(%)	1.6	1.4	0.8	1.00–1.80	0.42–4.49
CD4+CD25+CD127-/CD4+	(%)	6.3	6.2	2.0	5.9–6.7	1.2–11.0
	(cells/μl)	42	39	18.0	34–43	10–103
CD4+CD45RA-/CD4+	(%)	66.0	65.3	13.6	56.5–75.0	29.5–98.3
	(cells/μl)	456	439	171	333–549	194–1141
CD4+CD45RA+/CD4+	(%)	37.7	36.0	13.8	25.0–43.5	1.7–70.5
	(cells/μl)	455	439	176.9	291–465	20–1141
CD4+CD45RA+CD62L+/CD4+	(%)	34.2	35.0	13.6	25.0–43.8	1.9–69.2
	(cells/μl)	247	228	155	141–324	14–1037
CD4+CD28+/CD4+	(%)	91.6	94.8	9.9	87.4–97.9	48.4–99.9
	(cells/μl)	635	606	226	477–770	175–1478
CD4+CD38+/CD4+	(%)	10.6	8.8	4.1	5.2–14.3	1.0–29.7
	(cells/μl)	75	59	35.1	37–98	4–286
CD4+HLA-DR+/CD4+	(%)	7.3	6.1	4.0	4.2–9.6	1.3–18.0
	(cells/μl)	51	49	30	25–67	8–229
CD4+CD38+HLA-DR+/CD4+	(%)	1.0	0.7	0.3	0.5–1.1	0.1–10.0
	(cells/μl)	6	5	2	3–8	0–40
CD4+PD-1+/CD4+	(%)	14.5	13.5	5.2	10.5–18.1	2.5–28.9
	(cells/μl)	99	93	38	63–129	14–257
CD4+Ki-67+/CD4+	(%)	0.8	0.7	0.5	0.3–1.2	0.1–2.3
	(cells/μl)	6	5	3	2–8	0–19
CD4+CD45RA+CD31+/CD4+	(%)	23.5	24.2	11.9	14.2–31.3	0.6–59.8
	(cells/μl)	170	157	120	83–229	4–896
CD8+CD45RA-/CD8+	(%)	48.7	47.3	15.4	35.8–60.8	19.4–85.3
	(cells/μl)	244	219	125	161–313	40–781
CD8+CD45RA+CD62L+/CD8+	(%)	27.4	24.7	16.4	13.8–39.6	1.6–71.2
	(cells/μl)	137	113	96	62–204	6–464
CD8+CD28+/CD8+	(%)	52.5	53.8	20.2	36.2–67.2	13.6–90.0
	(cells/μl)	254	240	122	165–320	45–789
CD8+CD38+/CD8+	(%)	4.6	4.0	3.6	2.0–5.7	0.3–19.0
	(cells/μl)	23	17	20	9–30	2–111
CD8+HLA-DR+/CD8+	(%)	12.9	10.7	7.9	6.8–18.0	2.2–33.1
	(cells/μl)	66	50	42	27–96	8–308
CD8+CD38+HLA-DR+/CD8+	(%)	1.9	1.7	1.2	0.9–2.5	0.1–6.0
	(cells/μl)	10	8	6	4–13	1–45
CD8+PD-1+/CD8+	(%)	13.7	14.1	5.5	9.5–16.5	3.6–30.5
	(cells/μl)	69	61	37	41–88	9–221
CD8+Ki-67+/CD8+	(%)	0.7	0.5	0.3	0.3–0.8	0.1–2.1
	(cells/μl)	3	2	1	1–4	1–11
CD19+CD38+/CD19+	(%)	17.8	15.7	10.5	9.3–24.0	2.5–49.9
	(cells/μl)	34	27	26	15–48	2–200
CD19+PD-1+/CD19+	(%)	2.3	1.7	1.5	0.9–3.1	0.2–6.4
	(cells/μl)	3	2	1	2–5	1–14
CD19+Ki-67+/CD19+	(%)	1.2	0.9	0.6	0.6–1.3	0.1–3.8
	(cells/μl)	2	2	1	1–3	1–7
CD16+CD56+CD38+/CD16+CD56+	(%)	42.9	43.5	19.3	28.4–58.7	8.6–82.1
	(cells/μl)	131	111	90	75–161	14–631
CD16+CD56+HLA-DR+/CD16+CD56+	(%)	6.3	4.5	3.5	2.1–9.0	0.8–20.9
	(cells/μl)	21	13	13	6–31	1–112
CD16+CD56+PD-1+/CD16+CD56+	(%)	1.6	0.8	0.7	0.3–1.7	0.1–7.5
	(cells/μl)	5	3	3	1–5	1–35
CD16+CD56+Ki-67+/CD16+CD56+	(%)	1.1	0.9	0.7	0.4–1.6	0.1–3.8
	(cells/μl)	3	2	3	1–4	1–20

### The relationship between lymphocyte subsets and aging

**Figure 2:**
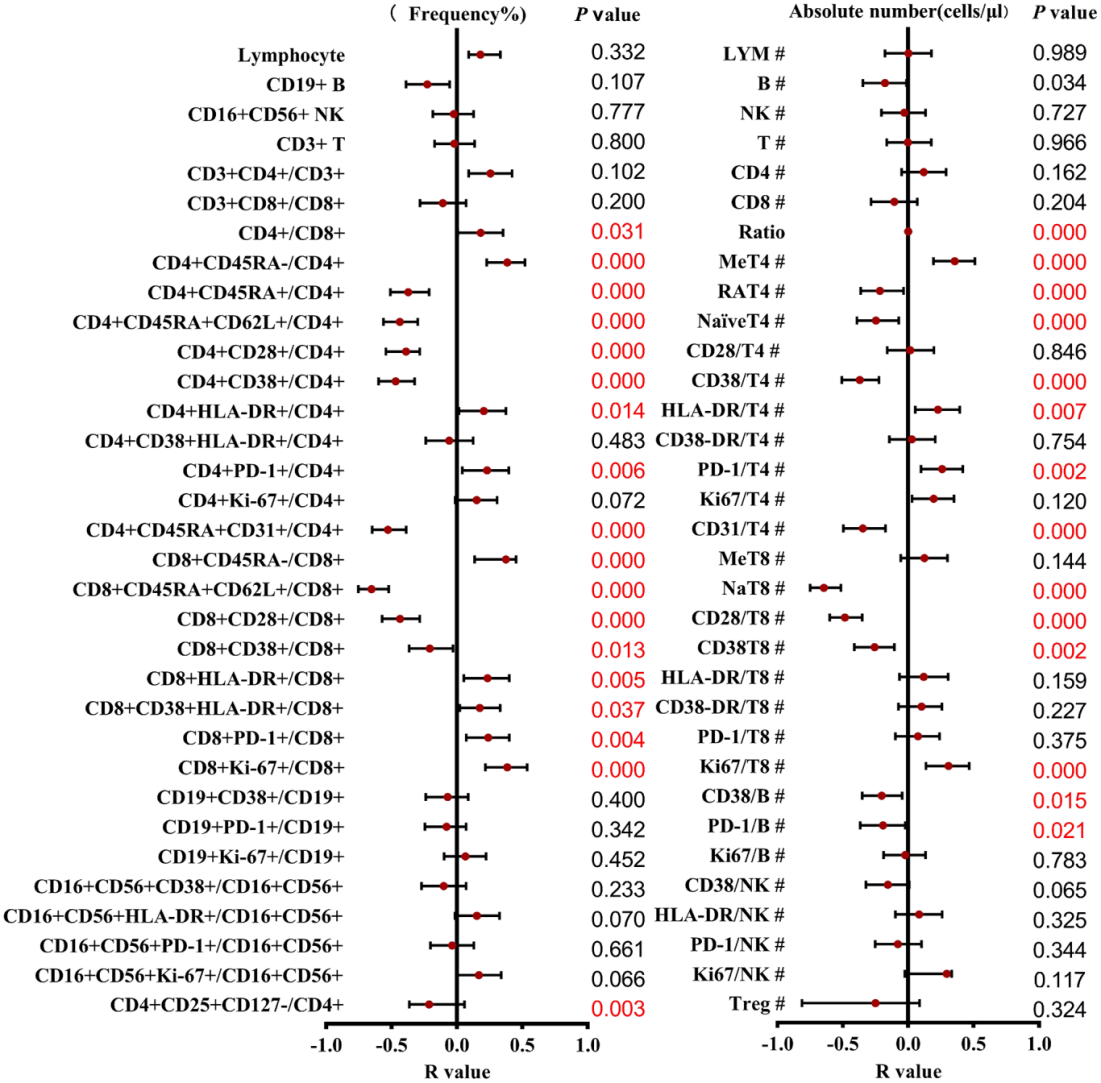
**Correlation and regression analysis of different T cell subsets and ages were calculated.** The left represents the frequency, and the right represents the absolute number. The red points and bars represent the R-value and 95% confidence interval of the regression equation, and the *P*-value to the right of the figure indicates the statistical significance of each subset.

To investigate the influence of the age on the lymphocyte subsets, we divided the subjects into three age groups. A total of 55 (36.6%) were in young adult group (19–44 years old, 34 males, 21 females, mean age 29.2 years), 46 (30.7%) belonged to the middle-aged adult group (45–64 years old, 24 males, 22 females, mean age 52.6 years) and 49 (32.7%) belonged to the elderly (over 65 years, 31 males, 18 females, mean age 68.0 years). The Chi-square tests showed that there were differences between the gender in different age groups. (*P* = 0.045). We then observed associations between a set of parameters and age (results shown in Figure 2). Most parameters varied with age except for CD19+ B cells (*p* = 0.304), CD16+CD56+ NK cells (*p* = 0.727), CD3+ T cells (*p* = 0.966), CD3+CD4+ T cells (*p* = 0.162) and CD3+CD8+T cells (*p* = 0.204), especially a clear association was shown in the CD4 and CD8 T cell subsets combinations. Gender impacts the fluctuation of many physiological parameters, furtherly, the lymphocyte subsets distributions correlated with age in males and females at different stages were evaluated and regression analysis was performed.

### T cell subsets decreased with older ages in male and female populations

**Figure 3:**
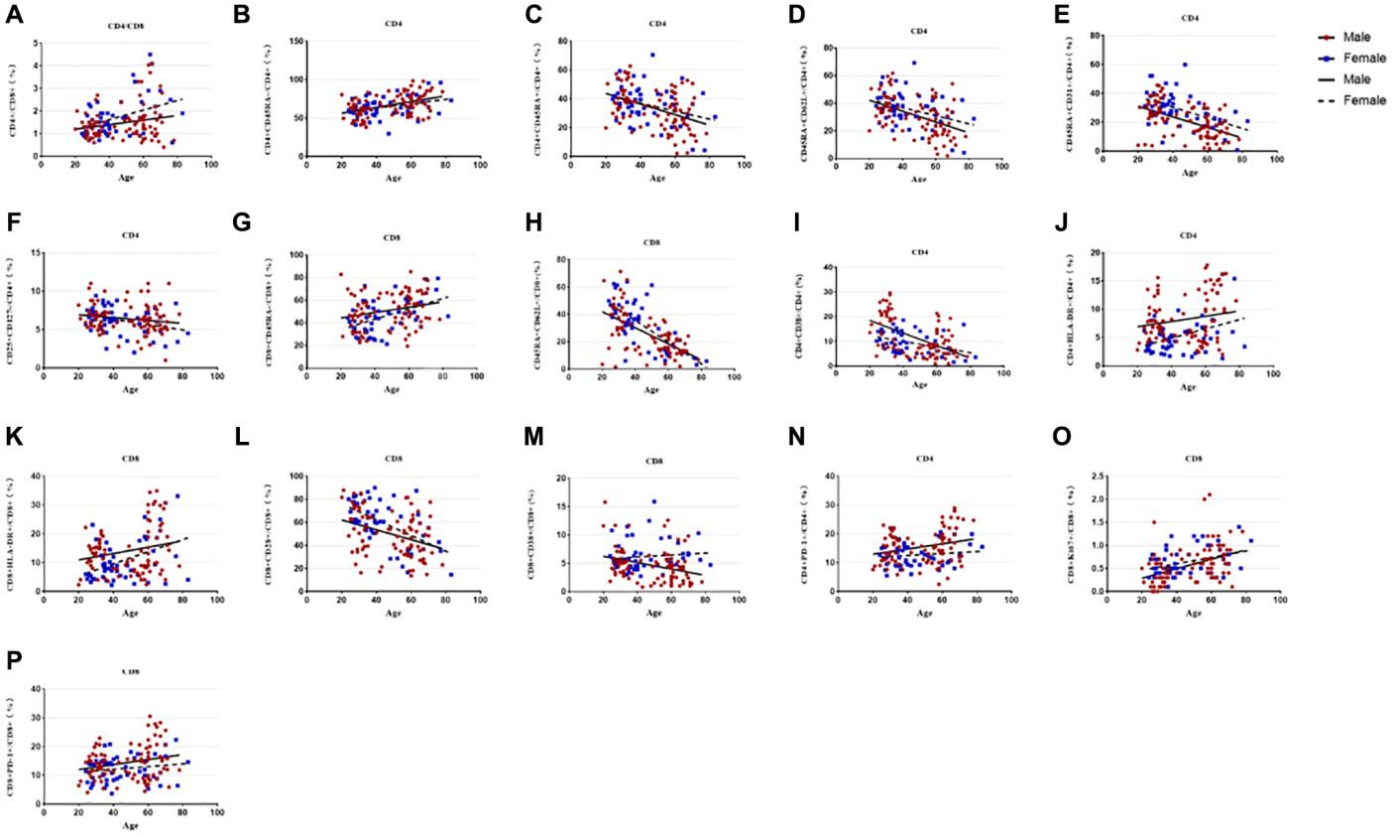
**Relationship between age and percentages of lymphocyte subsets in the male or female population.** (**A**) CD4+/CD8+ percentage, (**B**) CD4+CD45RA- T cell percentage, (**C**) CD4+CD45RA+ T cell percentage, (**D**) CD4+Naïve T cell percentage, (**E**) CD4+CD45RA+CD31+T cell percentage, (**F**) CD4+CD25+CD 127- T cell percentage, (**G**)CD8+CD45RA- T cell percentage, (**H**) CD8+Naive T cell percentage, (**I**) CD4+CD38+ T cell percentage, (**J**) CD4+HLA-DR+ T cell percentage, (**K**) CD8+HLA-DR+ T cell percentage, (**L**) CD8+CD28+ T cell percentage, (**M**) CD8+CD38+ T cell percentage, (**N**) CD4+PD-1+ T cell percentage, (**O**) CD8+Ki67+ T cell percentage, (**P**) CD8+PD-1+ T cell percentage. Correlations between two variables were done using Spearman’s correlation and linear regression was used to plot graph.

**Figure 4:**
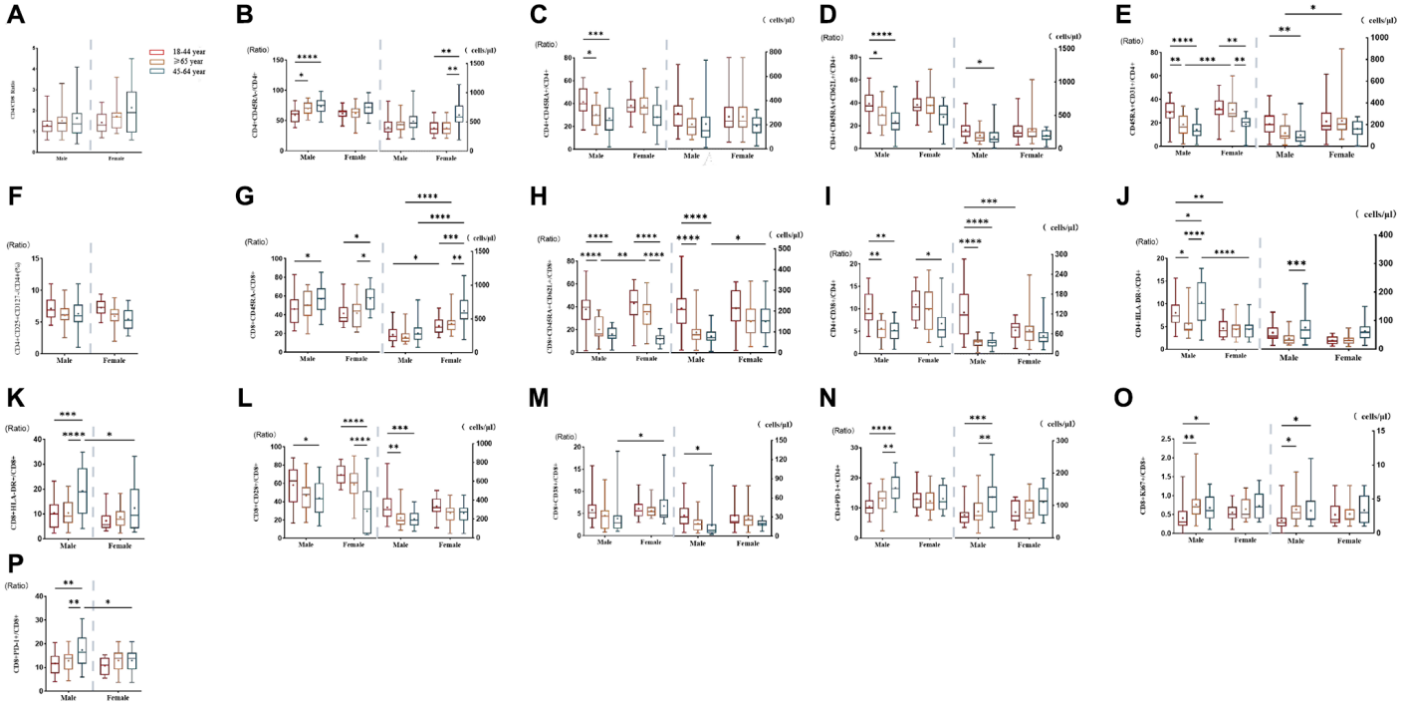
**Comparison of lymphocyte subsets in different age groups of male or female populations with significant differences.** (**A**) CD4+/CD8+percentage (%), (**B**) CD4+CD45RA- T cell (% and cells/μl), (**C**) CD4+CD45RA+ T cell (% and cells/μl), (**D**) CD4+ Naïve T cell (% and cells/μl), (**E**) CD4+CD45RA+CD31+T cell (% and cells/μl), (**F**) CD4+CD25+CD127- T cell percentage (%), (**G**) CD8+CD45RA- T cell (% and cells/μl), (**H**) CD8+Naive T cell (% and cells/μl), (**I**) CD4+CD38+ T cell (% and cells/μl), (**J**) CD4+HLA- DR+ T cell (% and cells/μl), (**K**) CD8+HLA-DR+ T cell (% and cells/μl), (**L**) CD8+CD28+ T cell (% and cells/μl), (**M**) CD8+CD38+ T cell (% and cells/μl), (**N**) CD4+PD-1+ T cell (% and cells/μl), (**O**) CD8+Ki67+ T cell (% and cells/μl), (**P**) CD8+PD-1+ T percentage (%). (^*^p < 0.05, ^**^p < 0.01, ^***^p < 0.001).

The results of subgroup distribution of males and females showed that CD4/CD8 percentage and the percentage of CD4+CD25+CD127-T cells had slightly increased or decreased trend with age (Figure 3A, 3F), whereas there was no significant difference among further groups (Figure 4A, 4F). Data showed a moderate decreased expression level of CD4+CD45RA+ T cells percentage (r = −0.303, *p* = 0.002, Figure 3C) and CD4+CD45RA+CD62L+ T cells percentage (r = −0.326, *p* < 0.001, Figure 3D) with age growing in males (Figure 4C, 4D). Meanwhile, it was found that aging did not seem to have a significant effect on women, but still showed an obvious downward trend. Significant differences existed in CD8+CD45RA+CD62L+ T cells and CD4+CD45RA+CD31+ T cells between the gender in the whole age stages and the difference mainly lay in males (Figure 4H, 4E). Figure 3E, 3H shows the strong negative correlation between these two types of cells with age (r = −0.598, *P* < 0.001 and r = −0.3379, *p* < 0.001). A trend of decrease in CD4+CD38+ T cells and CD8+CD38+ T cells were also observed with age (r = −0.446, *P* < 0.001 and r = −0.200, *P* < 0.001, Figure 3I, 3M), but there was only obvious difference with age growing in the males (Figure 4I). Differences in the percentage of CD8+CD38+ T cells mainly lay in the older group, and women were higher than men (Figure 4M). The same changes can also be observed in CD8+CD28+ T cells (Figure 4L), with a strong negative correlation in the CD8+CD28+ T percentage (r = −0.456, *P* < 0.001, Figure 3L), particularly evident in females.

### T cell subsets increased significantly with age between male and female individuals

Compared with CD45RA+ T cells, the reverse trend between CD4+CD45RA-T cells (r = 0.251, *p* = 0.001, Figure 3B) and CD8+CD45RA-T cells (r = 0.258, *p* = 0.018, Figure 3G) were also shown in our study which gradually increased with age (Figure 4B, 4G). Significant gender difference was found among three cohorts respectively in memory CD8+ T cells and were higher in women (Figure 4G). Data showed an increased expression level of CD4+HLA-DR+ T cells (r = −0.254, *p* = 0.002, Figure 3J) and the percentage of CD8+HLA-DR+ T cells (r = −0.226, *p* < 0.001, Figure 3K) with age growing. Meanwhile, it was found that age seem to have a significant effect on men between the older and the younger, and that men were significantly higher than women in the older group (Figure 4J). The same changes could be seen in CD8+HLA-DR+ percentage (Figure 4K). More interestingly, we found a weak positive correlation between age and CD4+PD-1+ T cells (r = 0.218, *p* = 0.016, Figure 3N), as well as CD8+PD-1+ percentage (r = 0.241, *p* < 0.001, Figure 3P). Age-related variation was obvious with CD4+PD-1+ T cells in the males (Figure 4N). Similarly, the percentage of CD8+PD-1+showed increased expression level with age growing in the males, and the gender difference was significant in the older group (Figure 4P). An increased trend of CD8+ki-67+ percentage was also observed with age (r = 0.201, *p* < 0.001, Figure 3O), and the obvious difference existed in men versus women towards the younger and the other older groups (Figure 4O).

## DISCUSSION

Immunosenescence is a concept that encompass all age-related changes in the immune system and describes the progressive and ubiquitous remodeling of immune function during aging [15]. Lymphocytes exert their immune function through activation, proliferation, differentiation, memory, exhaustion, and other mechanisms.[16]. Different subgroups exhibit unique trends and age-related immune function turning points [17, 18]. Therefore, age-related immune subgroup transformation and sex differences need to be further explored. We firstly conducted extensive flow cytometric analysis on peripheral blood from 150 healthy adults of all ages, depicting a series of age-related immune aging indicators, especially some emerging indicators of depletion and proliferation with potential clinical application merits, such as PD-1 and Ki67, which can be used as new markers for immunosenescence research. It provides a richer perspective on immune aging of peripheral blood lymphocyte subgroups in the elderly.

Our team conducted an observational study of 1068 individuals as early as 2016 to explore clues for immune aging [9]. Our research not only validated previous classic indicators, but also further evaluated the impact of aging on the quantity and phenotype of PD-1 and Ki67. At the same time, there are different immune aging patterns in terms of gender differences, and immune parameters of different ages and genders may have an impact on the design of immunotherapy for the elderly. Controversial findings regarding the effect of age on immune markers in peripheral blood have been reported [19–26]. Our results did not show any significant quantitative alteration in the overall T cells, NK cells, and B cells with age. Report of changes in Treg with age are conflicting [26]. The results may be inconsistent due to different characteristic markers (such as CD127− or Foxp3+). The conclusions drawn from different studies cannot be unified, possibly due to differences in inclusion and exclusion criteria, sample sizes etc, resulting in different trends in the data. Consistent with previous studies [4, 9, 15], our data also confirm a clear trend of reversal of age-related naïve T cells and memory cells, especially in the CD8 population, which is attributed to long-term repeated exposure to antigens and decreased thymic function [1, 8, 27]. The same changes could be seen in CD4+CD45RA+CD31+ T cells. The expression of CD31 is very low in most elderly people [28, 29], which reflects the new output capacity of thymus to a certain extent. Similarly to previous reports [9, 22, 25, 30–32], we also confirmed changes in different activation markers, with a significant increase in HLA-DR+ T cells and an opposite trend in early CD38+ T cells. In addition, we made a new discovery that the impact of aging is more pronounced in males, both in terms of percentage and absolute numbers, perhaps suggesting that older men are more susceptible to age-related diseases could potentially explain differences in lifespan between genders [33].

Depletion and senescence are two states of impaired T cell function, and recent studies have revealed the functional state of T cell failure, which is not an inert and non-functional state, but T cells showing a residual level of dysfunction [34]. The results showed that CD4+PD-1+ T cells and CD8+ PD-1+ T cells increased with age, and were more prominent in men. The gender difference is more obvious in the older group, showing that men are higher than women. Although little research has been done on the relationship between PD-1 and age, they have also shown increased expression of these proteins in older age groups, which is consistent with the characteristics of aging that we have developed. PD-1 is involved in the regulation of CD8 T-cell exhaustion during chronic viral infection and it is also transiently expressed by activated CD8 T cells during the acute phase of viral infection [35, 36]. Part of the research focused on mice [17, 36, 37], where their research found that the accumulation of PD-1+ memory type CD4+ T cell subsets gradually increased with age and dominated the normal mouse aging phase. Notably, in healthy individuals, most PD-1 expressing cells exhibit an effector memory phenotype rather than exhaustion phenotype in CD8+ T cells. The up-regulation of PD-1 on activated CD4+ and CD8+ T cells may contribute to differentiation and homeostasis of activated T cells, resulting in apoptosis and growth restriction [38]. In recent years, immunosenescence has received more and more attention due to its role in tumor development [38–42]. Due to the outcomes of immunotherapy are not consistent between elderly and young patients, with controversies surrounding the relationship between age and immune-related adverse events. Therefore, analyzing the latest data on the efficacy of immunotherapy in elderly cancer patients is crucial.

Ki-67 is a proliferating nuclear antigen that is associated with the mitotic cycle of cells and is often mentioned in cancer studies as an indicator of malignant proliferative activity [40, 43]. Our results also found that the Ki67+CD8+ percentage increased with age, and there was a significant difference between the younger and the older group. Studies have found that the proportion of Ki-67+ in peripheral blood increases with age, and CD8+ T cells proliferate more actively during aging [44]. There is not much literature support for the relationship between Ki-67 and age, so the specific reasons need to be further explored. Ki-67 is commonly used in pathologic diagnosis and is an independent predictor of breast cancer recurrence and survival [43]. Studies on the subgroup of lymphomas caused by fever of unknown origin, our team found that PD-1+ T cells were significantly higher in patients with fever and confirmed histologically than in non-tumor patients. PD-1 expression on effector T cells depletes anti-tumor immune function and impairs control of tumor growth [45]. In addition, the Ki67+ T cells in these patients were significantly increased in both percentage and absolute number, showing a strong proliferation of T cells. These patients generally undergo invasive tissue biopsy first, and the pathological results indicated neoplastic proliferation of lymphocytes or histiocytes, which was highly consistent with our peripheral blood response proliferation results. The results of the study have not yet been published. For Ki67 research, only a small amount of peripheral blood is required, with a short reporting time and high consistency with pathological results, making it very suitable for routine projects. At the same time, our team also prospectively explored the immunological, hematological profiles inducing lymphocyte subsets related to SARS-CoV-2 infection during the acute omicron epidemic abrupted in 2023 [46]. For elderly patients infected with SARS-CoV-2, severe COVID-19 has the characteristics of irreversible reduction, continuous activation and proliferation of NK cells and CD8+T cells, which is helpful for clinicians to identify and rescue severe or critically ill patients at an early stage.

## CONCLUSION

In short, immune parameters are different between young and old populations. We used flow cytometry immunophenotyping to evaluate the counts and percentages of circulating lymphocyte subpopulation in healthy young and elderly individuals, not only validating the reliability of classical aging markers but also discovering an increasing trend of PD-1 on T cell and Ki67 on CD8+ T cell along with aging, providing clues for further research on immunosenescence. These data contribute to a deeper understanding of immune aging. Gender and age have significant impacts on lymphocyte subsets, thus a systematic investigation of various interacting factors related to age-associated immune changes is necessary.

### Limitation

Although our enrollments are derived from the same hospital, with a certain diversity in population composition and immune indicators, there are still limitations in sample size and representativeness. However, the reliable mapping of human T cells across different generations is reliable. It is possible to further increase the sample size, integrate relevant research results through multi-center studies, and provide more accurate evidence for the dynamic analysis of immune aging-related cells through Meta-analysis.
